# Supplementary material for: GPI-anchored FGF directs cytoneme-mediated bidirectional contacts to regulate its tissue-specific dispersion
Source: Nat Commun. 2022 Jun 16;13:3482. doi: 10.1038/s41467-022-30417-1 (PMC9203819; doi:10.1038/s41467-022-30417-1)
Supplement: Supplementary file 3 — Description of Additional Supplementary Files [file 41467_2022_30417_MOESM3_ESM.docx]

**Description of Additional Supplementary Files**

**Supplementary Movie 1. Polarized cytonemes from the basal surface of disc *bnl-*source extended toward the overlaying ASP.** 3D-rendered views showing the spatial organization of the ASP epithelial cells (*btl>nls-GFP*) and vertical cytonemes emanating from the basal sides of the *bnl-*source (*bnl>mCherryCAAX*). Scale bar, 30 μm.

**Supplementary Movie 2. Repeated contact assembly and disassembly between ASP and Bnl-source cytonemes at the ASP:source interface.** Time-lapse movie with merged channel on the left and split red channel on the right. ASP: *btlGal4>CD8:*GFP; *bnl*-source: *bnlLexA>mCherryCAAX*.

**Supplementary Movie 3. CAM-like trans-pairing of S2-Bnl:GFP with S2-Btl:Cherry leading to the cell-cell adhesion.** 3D-rendered views showing synaptic trans-paring of a Bnl:GFP-expressing S2 cell and a Btl:Cherry-expressing S2 cell by forming a disc-like Bnl:GFP and Btl:Cherry co-clusters at the synaptic contact site.

Please ensure italics and for genotypes, and micrometer symbols are retained.

**LEGENDS:**

**Supplementary Movie 1. Polarized cytonemes from the basal surface of disc *bnl-*source extended toward the overlaying ASP.** 3D-rendered views showing the spatial organization of the ASP epithelial cells (*btl>nls-GFP*) and vertical cytonemes emanating from the basal sides of the *bnl-*source (*bnl>mCherryCAAX*). Scale bar, 30 μm.

**Supplementary Movie 2. Repeated contact assembly and disassembly between ASP and Bnl-source cytonemes at the ASP:source interface.** Time-lapse movie with merged channel on the left and split red channel on the right. ASP: *btlGal4>CD8:*GFP; *bnl*-source: *bnlLexA>mCherryCAAX*.

**Supplementary Movie 3. CAM-like trans-pairing of S2-Bnl:GFP with S2-Btl:Cherry leading to the cell-cell adhesion.** 3D-rendered views showing synaptic trans-paring of a Bnl:GFP-expressing S2 cell and a Btl:Cherry-expressing S2 cell by forming a disc-like Bnl:GFP and Btl:Cherry co-clusters at the synaptic contact site.

**Supplementary Movies 4 and 5. Localization of f-actin together with Bnl:GFP and Btl:Cherry at the adhesive interface of trans-paired S2-Bnl:GFP::S2-Btl:Cherry.** 3D-rendered views of trans-paired S2-Bnl:GFP and S2:Btl:Cherry, showing the disc-like enrichment of f-actin (blue, stained with Phalloidin-Alexa 647) at the cell-cell synaptic interface. Movie 5 shows split blue channel (phalloidin) side by side.

**Supplementary Movie 6. Increased rate of ASP cytonemes’ extension/retraction when *bnl*-source cells overexpressed Bnl:GFP.** Time-lapse movie showing only ASP cytonemes (red, *btl>mCherryCAAX*); Bnl:GFP channel not shown. See also Supplementary Table 1.

**Supplementary Movie 7. Long polarized Bnl:GFPΔC-TM-presenting source cytonemes adhering to the ASP.** 3D-rendered views of Figure 6k, showing that the mCherryCAAX-marked disc *bnl-*source expressing Bnl:GFPΔC-TM extends long polarized cytonemes to adhere to the ASP (unmarked) with Bnl:GFPΔC-TM puncta at ASP:cytonemes contacts sites.

**Supplementary Movies 8 and 9. Bnl:GFPΔC-TM accumulation at multiple inter-cytoneme contact sites between the ASP and source.** Disc source cells: *bnl>CD4:IF2,Bnl:GFP*ΔC*-TM* (blue); ASP: *btl>mCherryCAAX*. Either 3D-rendered views (Supplementary Movie 8) or Serial z-sections (Supplementary Movie 9) highlighted cytoneme:cytoneme adhesion between the source and the ASP and enrichment of Bnl:GFPΔC-TM puncta at multiple contact sites. See also Figure 6j.

**Supplementary Movie 10. An increase in source cytoneme lifetime when Bnl:GFPΔC-TM was expressed from the source.** Time lapse movie showing Bnl:GFPΔC-TM-expressing source cytoneme (red, *bnl>mCherryCAAX*,Bnl:GFPΔC-TM) dynamics. Recipient cytonemes are unmarked, but are adhered to the source cytonemes. Time interval, 5 min.

**Supplementary Movie 11. Increase in adhesive affinity and contact stability of Bnl:GFPΔC-TM-exchanging ASP and source cytonemes leads to bidirectional force and cytoneme breakage.** ASP: *btl>mCherryCAAX* (blue); *bnl-*source: *bnl>CD4:IFP2* (red), Bnl:GFPΔC-TM. Time-lapse movie showing stable contact-dependent interactions between ASP and Bnl:GFPΔC-TM-expressing source. Note the stabilized source and recipient cytonemes bidirectionally pull and stretch each other, leading to cytoneme breakage, unlike *wt* (Supplementary Movie 2). Time interval, 10 min.

**Supplementary Movie 12. ASP-specific dispersion of Bnl:GFP from the wing disc *bnl-*source.** 3D-rendered views of the wing disc with Bnl:GFP overexpressed in the source (red, *UAS-mCherryCAAX;bnl-Gal4* X *UAS-Bnl:GFP*) showing target-specific long-range Bnl dispersion; cell outlines marked with Phalloidin Alexa-Fluor 647, blue; larger ASP cells are more intensely stained and in-focus than the underlaying wing disc cells. Scale bar, 20 μm between major tick marks.

**Supplementary Movies 13 and 14. Non-specific distribution of Bnl:GFPΔC variants.** 3D-rendered views of the wing disc with Bnl:GFPΔC_168_ overexpressed in the source (red, *UAS-mCherryCAAX;bnl-Gal4* X *UAS-Bnl:GFP*Δ*C_168_*) showing the non-specific dispersion of the signal to surrounding wing disc cells. Cell outlines marked with Phalloidin Alexa-Fluor 647, blue. Scale bars, 20 μm between major tick marks.

**Supplementary Movies 15 and 16. Restricted dispersion of Bnl:GFPΔC-TM in the ASP.** 3D-rendered views of the wing disc with Bnl:GFP**Δ**C_40_-TM (Movie 15) Bnl:GFP**Δ**C_168_-TM (Movie 16) overexpressed in the source (red, *UAS-mCherryCAAX;bnl-Gal4* X *UAS-Bnl:GFP***Δ***C_168_-TM* ) showing target-specific but restricted dispersion of the signal to only the tip of ASP cells. Note that the signal received by ASP cells colocalized with source cell membrane (GFP + Cherry signals); cell outlines marked with Phalloidin Alexa-Fluor 647, blue. Scale bar, 20 μm between major ticks.
