# Supplementary material for: GPI-anchored FGF directs cytoneme-mediated bidirectional contacts to regulate its tissue-specific dispersion
Source: Nat Commun. 2022 Jun 16;13:3482. doi: 10.1038/s41467-022-30417-1 (PMC9203819; doi:10.1038/s41467-022-30417-1)
Supplement: Supplementary file 20 — Reporting Summary [file 41467_2022_30417_MOESM20_ESM.pdf]

nature portfolio

Corresponding author(s): Sougata Roy

Last updated by author(s): Feb 9, 2022

Reporting Summary

Nature Portfolio wishes to improve the reproducibility of the work that we publish. This form provides structure for consistency and transparency in reporting. For further information on Nature Portfolio policies, see our Editorial Policies and the Editorial Policy Checklist.

Statistics

For all statistical analyses, confirm that the following items are present in the figure legend, table legend, main text, or Methods section.

n/a	Confirmed	
☐	☒	The exact sample size (n) for each experimental group/condition, given as a discrete number and unit of measurement
☐	☒	A statement on whether measurements were taken from distinct samples or whether the same sample was measured repeatedly
☐	☒	The statistical test(s) used AND whether they are one- or two-sided<br>*Only common tests should be described solely by name; describe more complex techniques in the Methods section.*
☒	☐	A description of all covariates tested
☒	☐	A description of any assumptions or corrections, such as tests of normality and adjustment for multiple comparisons
☐	☒	A full description of the statistical parameters including central tendency (e.g. means) or other basic estimates (e.g. regression coefficient) AND variation (e.g. standard deviation) or associated estimates of uncertainty (e.g. confidence intervals)
☐	☒	For null hypothesis testing, the test statistic (e.g. F, t, r) with confidence intervals, effect sizes, degrees of freedom and P value noted<br>*Give P values as exact values whenever suitable.*
☒	☐	For Bayesian analysis, information on the choice of priors and Markov chain Monte Carlo settings
☒	☐	For hierarchical and complex designs, identification of the appropriate level for tests and full reporting of outcomes
☒	☐	Estimates of effect sizes (e.g. Cohen's d, Pearson's r), indicating how they were calculated

Our web collection on statistics for biologists contains articles on many of the points above.

Software and code

Policy information about availability of computer code

Data collection	Image data were visualized and analyzed in Andor iQ3 (for Yokogawa CSUX1 spinning disc confocal), in Zen 3 for Zeiss LSM900/Aireyscan 2; FIJI/ImageJ2 2.3.0/1.53f (https://fiji.sc) and Imaris 9.5.0 (Bitplane AG) were used for image processing, 3D views, and making movies. Sequence data were visualized and analyzed with SnapGene 3.3.4 and MacVector 13.5. Adobe Photoshop 22.5.1 and Adobe Illustrator 25.4.1 were used to assemble figure panels. Flow cytometry data were collected and analyzed in FACSDiva (BD Biosciences) v6.1.3.
Data analysis	Statistical analyses were performed using VassarStat (vassarstats.net) and GraphPad Prism 8, MS Excel version 16.30. P values were determined either using the unpaired two-tailed t test for pair-wise comparisons or the one-way ANOVA followed by Tukey's honestly significant different (HSD) test for comparison of multiple groups. The sample size (N) for each data analysis is indicated in the figures/figure legends/methods/source data. P and r values, range of distribution of numerical data and standard deviations are either shown in the respective Figure panels/Tables or are described in the figure legends, methods, and source data files. Rose plots were generated by ggplot2 plot builder using R software version 3.6.2. Sholl analysis was performed using FIJI/ImageJ2 2.3.0/1.53f (https://fiji.sc). Protein sequence analyses were done using ProtScale (ExPASy: https://web.expasy.org/protscale/), EMBOSS Pepinfo (www.ebi.ac.uk), PredGPI (http://gpcr.biocomp.unibo.it/predgpi).

For manuscripts utilizing custom algorithms or software that are central to the research but not yet described in published literature, software must be made available to editors and reviewers. We strongly encourage code deposition in a community repository (e.g. GitHub). See the Nature Portfolio guidelines for submitting code & software for further information.

Data

Policy information about availability of data

All manuscripts must include a data availability statement. This statement should provide the following information, where applicable:

- Accession codes, unique identifiers, or web links for publicly available datasets
- A description of any restrictions on data availability
- For clinical datasets or third party data, please ensure that the statement adheres to our policy

All data generated and analyzed are included in the manuscript and supporting files. Source data are provided with this paper.

Field-specific reporting

Please select the one below that is the best fit for your research. If you are not sure, read the appropriate sections before making your selection.

[x] Life sciences [ ] Behavioural & social sciences [ ] Ecological, evolutionary & environmental sciences

For a reference copy of the document with all sections, see nature.com/documents/nr-reporting-summary-flat.pdf

Life sciences study design

All studies must disclose on these points even when the disclosure is negative.

Sample size	No statistical method was used to predetermine sample size. The sample size was based on the current standard in the field as well as previous experimental experience. The information about the number of independent replicates, sample size, methods of group allocation, and statistical analyses are mentioned in the main text, methods, figure legends, supplementary tables and Source Data.
Data exclusions	No data were excluded.
Replication	The number of independent replicates are mentioned in the texts, Figure/Table legends, and Materials and Methods sections. At least three independent experimental replicates were used.
Randomization	Random samples were selected for each group and the number of random samples used is mentioned in the Figures/Tables/Methods/Texts. Same genetic background was used to compare between the control and the test animals. Mosaic analyses include control and test areas within the same organ of an animal. The number of independent animals used for validating the data were mentioned in the relevant text, Figures/tables. The methods section, figure/table legends, and the texts describe the group allocation.
Blinding	In all genetic experiments, the genotype needed to be determined based on different fly genetic/chromosome markers, so blinding was not employed. Samples were selected randomly and unbiased manner. For the biochemical experiments, different reagents for the particular data set needed to be added and then analyzed using specific technical approaches, and so blinding was not employed. For comparative analyses, internal control and normalized values were compared in an unbiased manner and differences between the control and experimental conditions were highly notable and reproducible in both biological and technical replicates.

Reporting for specific materials, systems and methods

We require information from authors about some types of materials, experimental systems and methods used in many studies. Here, indicate whether each material, system or method listed is relevant to your study. If you are not sure if a list item applies to your research, read the appropriate section before selecting a response.

Materials & experimental systems

n/a	Involved in the study	
[ ]	[x]	Antibodies
[ ]	[x]	Eukaryotic cell lines
[x]	[ ]	Palaeontology and archaeology
[ ]	[x]	Animals and other organisms
[x]	[ ]	Human research participants
[x]	[ ]	Clinical data
[x]	[ ]	Dual use research of concern

Methods

n/a	Involved in the study	
[x]	[ ]	ChIP-seq
[ ]	[x]	Flow cytometry
[x]	[ ]	MRI-based neuroimaging

Antibodies

Antibodies used	Mouse monoclonal anti-Discs large (Dlg) (1:100; DSHB, Cat# 4F3 anti-discs large; RRID: AB_528203); Phospho-p44/42 MAPK (Erk1/2) (Thr202/Tyr204) rabbit monoclonal antibody (dpERK) (1:250 in tissue and 1:1000 in S2 cells; Cell signaling Technology; Cat# 4370; RRID: AB_2315112); Rat monoclonal anti-HA (3F10) (1:1000 for standard and 1:500 for EIF; Roche; Cat#11867423001; RRID:

AB_390918); Rabbit polyclonal anti-Bnl (1:500 for EIF, created in our previous study: eLife 2018;7:e38137); Rabbit anti-GFP antibody (1:3000 for EIF; Abcam; Cat# ab6556; RRID: AB_305564); Goat anti-Mouse IgG (H+L) Alexa Fluor 555 (1:1000; Thermo Fisher Scientific; A21434); Goat anti-Mouse IgG (H+L) Alexa Fluor 647 (1:1000; Thermo Fisher Scientific; A28181); Goat anti-Rat IgG (H+L) Alexa Fluor 647 (1:1000; Thermo Fisher Scientific; A21247); Goat anti-Rabbit IgG (H+L) Alexa Fluor 555 (1:1000; Thermo Fisher Scientific; A21428); Goat anti-Rabbit IgG (H+L) Alexa Fluor 647 (1:1000; Thermo Fisher Scientific; A21244). All the information is provided in Supplementary Table 4.

Validation

Rabbit polyclonal anti-Bnl antibody was validated by IHC and published in eLife 2018;7:e38137. Validation statement: "To ensure that the GFP-marked puncta in the ASP were actual Bnl molecules, we performed an immunohistochemistry assay (IHC) with αBnl antibody on the homozygous bnl:gfpendo larval tissues. The Bnl antibody recognized all of the Bnl:GFP puncta in the larval ASP, confirming that they represent the Bnl protein."

The following antibodies are from commercial sources and have been validated by the vendors:
(1) Mouse monoclonal anti-Discs large (Dlg) (1:100; DSHB, Cat# 4F3 anti-discs large; RRID: AB_528203)
(a) Vendor website: https://dshb.biology.uiowa.edu/4F3-anti-discs-large
(b) Vendor Validation statement: https://dshb.biology.uiowa.edu/core/media/media.nl?id=1523553&c=571578&h=O22gYwGyD6-6QmGzY5i_W20gahwIwy1BpZJrZUXkQ2mjAtd1&_xt=.pdf
(c) Validation methods: IHC, IP, Western blot; Reactivity: Drosophila melanogaster.

(2) Phospho-p44/42 MAPK (Erk1/2) (Thr202/Tyr204) rabbit monoclonal antibody (dpERK) (1:250 in tissue and 1:1000 in S2 cells; Cell signaling Technology; Cat# 4370; RRID: AB_2315112)
(a) Vendor website: https://www.cellsignal.com/products/primary-antibodies/phospho-p44-42-mapk-erk1-2-thr202-tyr204-d13-14-4e-xp-rabbit-mab/4370
(b) Vendor Validation statement: Phospho-p44/42 MAPK (Erk1/2) (Thr202/Tyr204) (D13.14.4E) XP® Rabbit mAb detects endogenous levels of p44 and p42 MAP Kinase (Erk1 and Erk2) when dually phosphorylated at Thr202 and Tyr204 of Erk1 (Thr185 and Tyr187 of Erk2), and singly phosphorylated at Thr202. This antibody does not cross-react with the corresponding phosphorylated residues of either JNK/SAPK or p38 MAP kinases.
(c) Validation methods: IHC, IP, Western blot, Flow Cytometry; Reactivity: Human, Mouse, Rat, Hamster, Monkey, Mink, D. melanogaster, Zebrafish, Bovine, Dog, Pig, S. cerevisiae.

(3) Rat monoclonal anti-HA (3F10) (1:1000 for standard and 1:500 for EIF; Roche; Cat#11867423001; RRID: AB_390918)
(a) Vendor website: https://www.sigmaaldrich.com/US/en/product/roche/roahaha
(b) Vendor Validation statement: Anti-HA High Affinity is a monoclonal antibody to the HA-peptide (clone 3F10). Anti-HA High Affinity recognizes the HA peptide sequence (YPYDVPDYA), derived from the influenza hemagglutinin protein. The antibody recognizes its antigenic determinant even when the HA peptide epitope is introduced into unrelated recombinant proteins by a technique known as "epitope tagging".
(c) Validation methods: IHC, IP, Western blot; Reactivity: Anti-HA, High Affinity recognizes the 9-amino acid sequence YPYDVPDYA, derived from the human influenza hemagglutinin (HA) protein. This epitope is also recognized in fusion proteins regardless of its position (N-terminal, C-terminal or internal).

(4) Rabbit anti-GFP antibody (1:3000 for EIF; Abcam; Cat# ab6556; RRID: AB_305564)
(a) Vendor website: https://www.abcam.com/gfp-antibody-ab6556.html
(b) Vendor Validation statement: This antibody (ab6556) is the purified version of our best-selling rabbit polyclonal to GFP (ab290). It has been developed specifically for use in applications requiring a high titre and specificity with minimum background such as immuno-electron microscopy. This anti-GFP antibody recognizes the enhanced form of GFP as well.
(c) Validation methods: IHC, IP, Western blot, Electron Microscopy, Flow Cytometry; Reactivity: Species independent; GFP antibody (ab6556) is reactive against all variants of Aequorea victoria GFP such as S65T-GFP, RS-GFP, YFP, CFP, RFP and EGFP.

Eukaryotic cell lines

Policy information about cell lines

Cell line source(s)	Drosophila S2-DGRC (Stock #6) from Drosophila Genomic Resource Center
Authentication	None of the cell lines used were authenticated
Mycoplasma contamination	Mycoplasma free cells used; mycoplasma contamination tested every six months
Commonly misidentified lines (See ICLAC register)	None used

Animals and other organisms

Policy information about studies involving animals; ARRIVE guidelines recommended for reporting animal research

Laboratory animals

Drosophila melanogaster male and female larvae and adults were used in this study. For genetic crosses, fresh female virgins (< 1 week old adults) were mated to males (1-2 weeks old adults), and their progeny were used for further analyses at the 3rd instar larval stages. Transgenic lines were produced using w1118 strain by P-element mediated germline transformation as described in Du et al., Elife, 2018. Transgenic injections were performed by Rainbow Transgenic Flies, Inc. Transgenic strains generated in this study: UAS-Bnl:GFP-deltaC, UAS-Bnl:GFP-deltaC-TM, UAS-Bnl:GFP-deltaC168, UAS-Bnl:GFP-deltaC168-TM, LexO-BtlDN:Cherry, UAS-Bnl:GFP-omega-m. Other strains and sources: Bloomington Drosophila Stock Center: UAS-CD8:GFP (5137), UAS-nls:GFP (4776), UAS-

mCherryCAAX (59021), UAS-CD4:mIFP (64182), UAS-Bnl (64232), UAS-diaRNAi (33424), hs-Flp (6), tub-Gal80ts (7108), act>CD2>Gal4 (4780), and w1118 (3605). Vienna Drosophila Resource Center: btl:GFP fTRG (318302). Kyoto Stock Center: bnl-Gal4 (112825). bnl:gfp-endo and btl:cherry-endo from Du et. al., eLife 2018;7:e38137; UAS-Bnl:GFP from Sohr et al. J Cell Biol. 2019 doi: 10.1083/jcb.201810138; lexO-mCherryCAAX, UAS-Dia-GFP, UAS-deltaDAD-Dia-GFP, btl-LHG from Roy et al., Science, 2014; UAS-BtlDN from Reichman-Fried & Shilo. Mech Develop 52, 265–273 (1995); btl-Gal4 from Sato & Kornberg. Dev Cell 3, 195–207 (2002); hs-FLP; btl>y+>Gal4,btl-mRFP1moe from Cabernard & Affolter. Dev Cell 9, 831–842 (2005).

Wild animals	The study did not involve wild animals.
Field-collected samples	The study did not involve field-collected samples.
Ethics oversight	All animal work was done following the University guidelines and the study did not require any additional ethical approval.

Note that full information on the approval of the study protocol must also be provided in the manuscript.

Flow Cytometry

Plots

Confirm that:

- [x] The axis labels state the marker and fluorochrome used (e.g. CD4-FITC).
- [x] The axis scales are clearly visible. Include numbers along axes only for bottom left plot of group (a 'group' is an analysis of identical markers).
- [x] All plots are contour plots with outliers or pseudocolor plots.
- [x] A numerical value for number of cells or percentage (with statistics) is provided.

Methodology

Sample preparation	Drosophila S2 cells were transfected following standard protocol and stained with detergent free immunostaining. After staining, the cells were resuspended in 600ul fresh PBS for flow cytometry.
Instrument	BD FACSCanto II
Software	BD FACSDiva v6.1.3
Cell population abundance	Each sample had at least 100,000 live singlets. Among all detected events, 50% were live cells, and 90% of the live cells were singlets. Live cells or singlets were determined according to FSC/SSC gating as described below in gating strategy.
Gating strategy	Cells were first gated to select live cells by selecting events with SSC less than 30000, FSC between 50 and 230. Within the live cell population, singlets are gated by selecting events showing proportional relation between FSC-A and FSC-H. Examples of Gating strategy presented in Supplementary data.

- [x] Tick this box to confirm that a figure exemplifying the gating strategy is provided in the Supplementary Information.
